# Supplementary material for: Genome-Wide Identification and Expression Analysis of the SWEET Gene Family in Annual Alfalfa (Medicago polymorpha)
Source: Plants (Basel). 2023 May 10;12(10):1948. doi: 10.3390/plants12101948 (PMC10222687; doi:10.3390/plants12101948)
Supplement: Supplementary file 1 [file plants-12-01948-s001.zip › Table S2..pdf]

Table S2. The information orthologous *SWEET* genes in *M. truncatula* and *M. polymorpha*

Gene name	Gene ID	Chr	Satart	End	Length	（bits）	E Value	Identities	Gaps
MpSWEET01	Medtr4g106990	chr4	43976500	43978913	2414	2109	0	95%	2%
MpSWEET02	Medtr8g096310	chr8	40382758	40385234	2477	974	0	91%	6%
MpSWEET03	Medtr8g096320	chr8	40403635	40405942	2308	780	0	91%	8%
MpSWEET04	Medtr8g099730	chr8	42223590	42226325	2736	1546	0	93%	3%
MpSWEET05	Medtr7g405710	chr7	224711	226577	1867	1535	0	92%	5%
MpSWEET06	Medtr7g405730	chr7	238597	240382	1786	1498	0	88%	8%
MpSWEET07	Medtr7g007490	chr7	1515108	1517630	2523	883	0	92%	3%
MpSWEET08	Medtr3g080990	chr3	36674946	36676295	1350	1369	0	90%	7%
MpSWEET09	Medtr3g089125	chr3	40831984	40834426	2443	1371	0	90%	4%
MpSWEET10	Medtr3g090940	chr3	41296423	41298378	1956	1766	0	88%	4%
MpSWEET11	Medtr3g090950	chr3	41306407	41308335	1929	1138	0	87%	6%
MpSWEET12	Medtr3g098910	chr3	45309861	45311769	1909	1014	0	91%	6%
MpSWEET13	Medtr3g098930	chr3	45314680	45319675	4996	1079	0	92%	3%
MpSWEET14	Medtr1g028460	chr1	9609945	9612620	2676	2283	0	91%	6%
MpSWEET15	Medtr1g029380	chr1	10054058	10056439	2382	1312	0	89%	6%
MpSWEET16	Medtr6g007623	chr6	1684113	1685864	1752	346	7.00E-94	90%	0%
MpSWEET17	Medtr6g007633	chr6	1694537	1696430	1894	521	9.00E-147	78%	8%
MpSWEET18	Medtr6g007637	chr6	1699177	1703592	4416	1339	0	87%	6%
MpSWEET20	Medtr2g007890	chr2	1184452	1186389	1938	2305	0	90%	6%
MpSWEET21	Medtr2g436310	chr2	14123315	14129655	6341	773	0	85%	8%
MpSWEET22	Medtr2g073190	chr2	30983345	30991272	7928	1114	0	90%	4%
MpSWEET23	Medtr5g067530	chr5	28543252	28545616	2365	1589	0	91%	2%
