# Supplementary material for: Genome-Wide Identification and Expression Analysis of the SWEET Gene Family in Annual Alfalfa (Medicago polymorpha)
Source: Plants (Basel). 2023 May 10;12(10):1948. doi: 10.3390/plants12101948 (PMC10222687; doi:10.3390/plants12101948)
Supplement: Supplementary file 1 [file plants-12-01948-s001.zip › Table S3.pdf]

Table S3. The information orthologous *SWEET* genes in *M*。*sativa* and *M. polymorpha*

Gene name	Gene ID	length	e-value	score	ident %
MpSWEET02	MsG0480023577.01	849	0	1136	83.5
MpSWEET03	MsG0480023578.01	2012	0	2406	91.4
MpSWEET05	MsG0780035928.01	908	0	1518	92.9
MpSWEET07	MsG0780036097.01	9022	0	2648	88.7
MpSWEET09	MsG0780040025.01	3407	6.4629E-07	64	85.2
MpSWEET10	MsG0380016494.01	4278	0	2330	88.9
MpSWEET12	MsG0380016975.01	11025	0	3434	92.2
MpSWEET14	MsG0180000993.01	2217	0	3250	87
MpSWEET18	MsG0680030472.01	2801	2.6421E-100	410	91.4
MpSWEET19	MsG0680030475.01	7304	6.262E-131	520	78.7
MpSWEET21	MsG0280007959.01	9501	0	2080	82.2
MpSWEET22	MsG0280009931.01	3703	0	1170	86.4
