# Supplementary figures and images for: Genome-Wide Identification and Expression Analysis of the SWEET Gene Family in Annual Alfalfa (Medicago polymorpha)
Source: Plants (Basel). 2023 May 10;12(10):1948. doi: 10.3390/plants12101948 (PMC10222687; doi:10.3390/plants12101948)

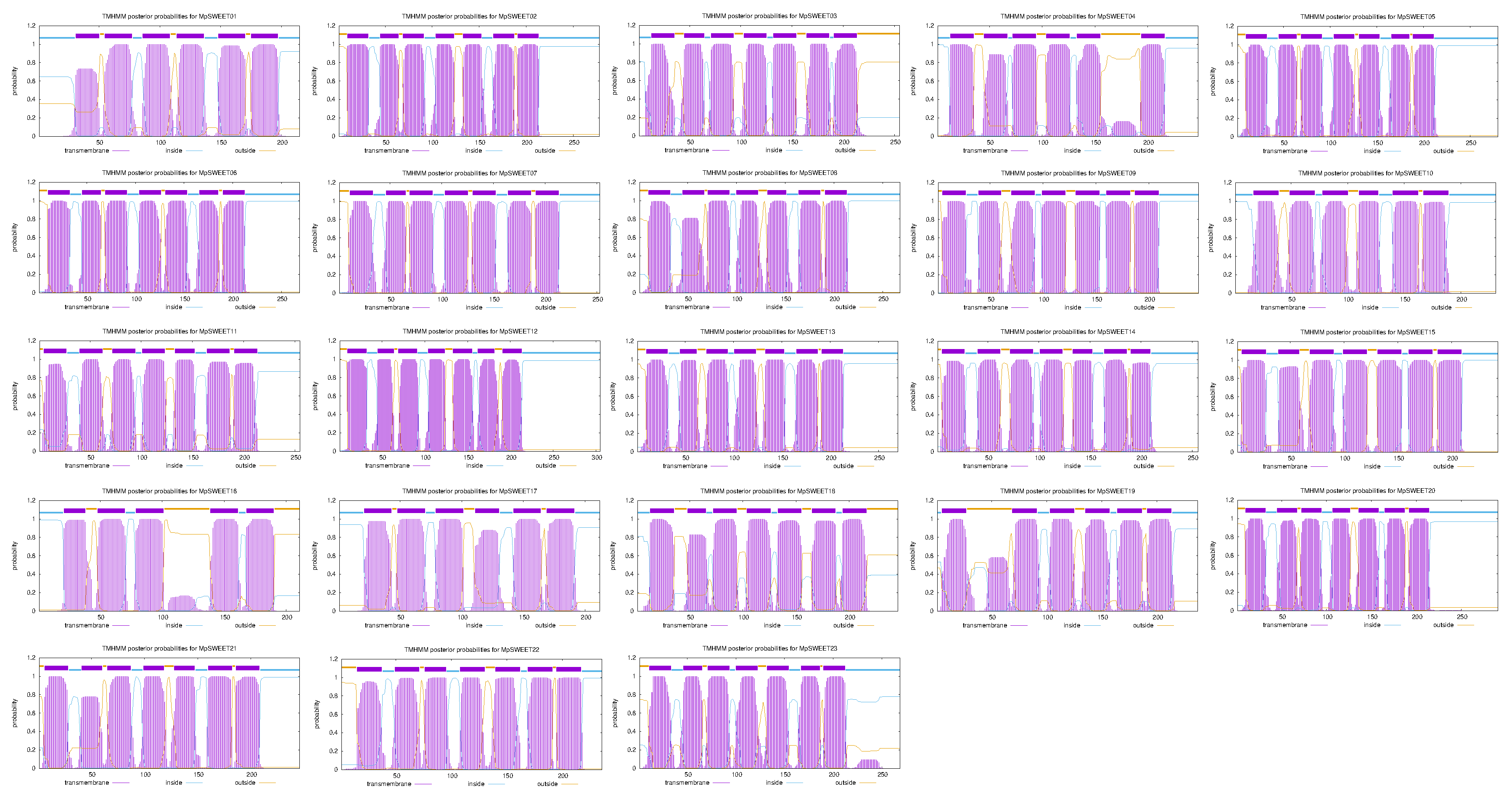

Supplement: Supplementary file 1 [file plants-12-01948-s001.zip › Figure S1..tif]

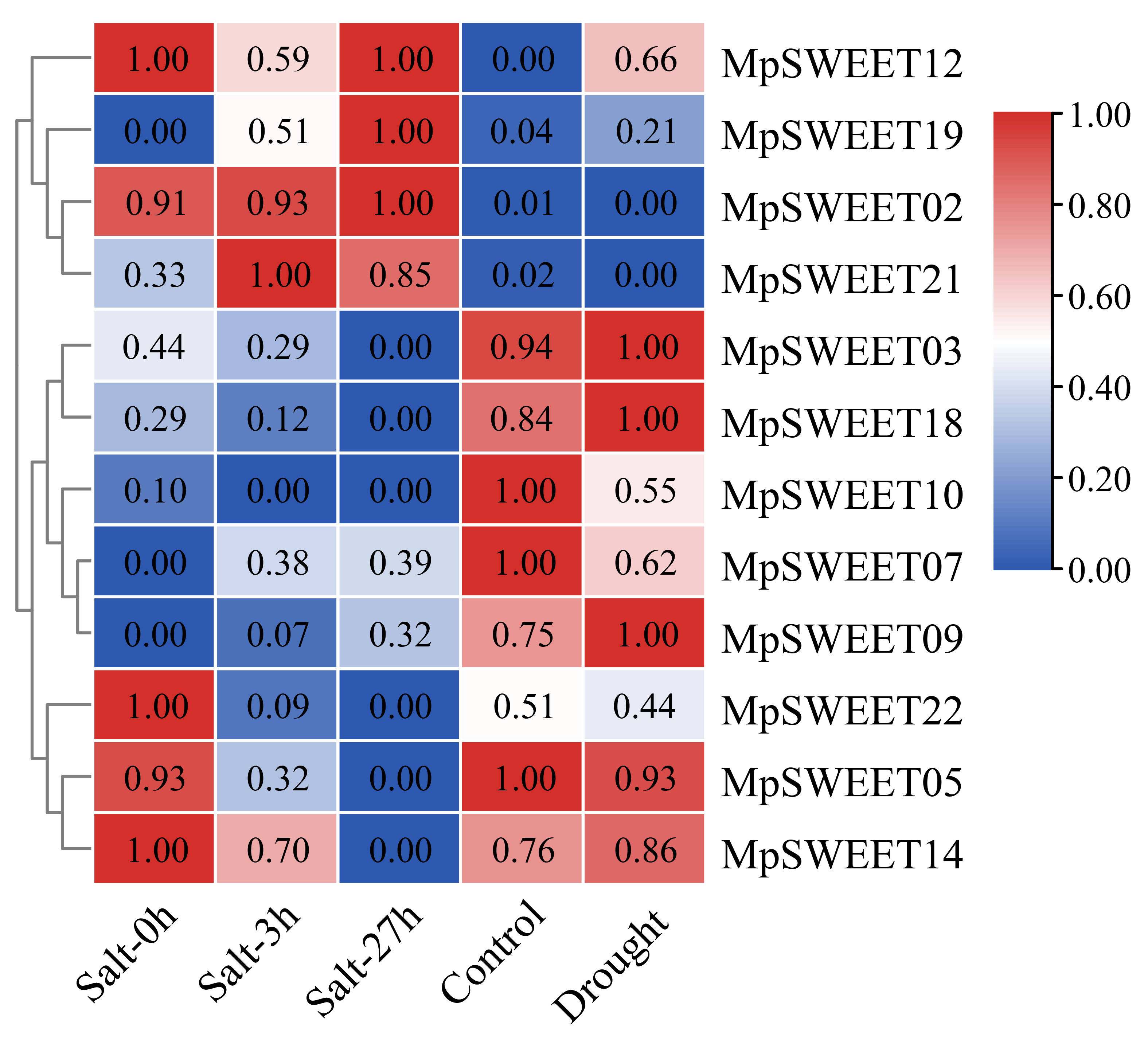

Supplement: Supplementary file 1 [file plants-12-01948-s001.zip › Figure S2..tif]

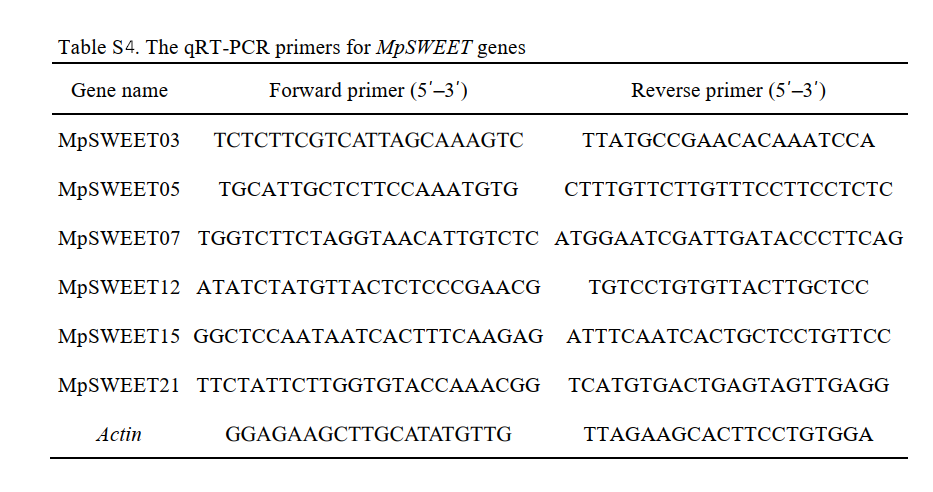

Supplement: Supplementary file 1 [file plants-12-01948-s001.zip › Table S4..png]
